# Supplementary material for: Taxonomy-guided selection of Paraburkholderia busanensis sp. nov.: a versatile biocontrol agent with mycophagy against Colletotrichum scovillei causing pepper anthracnose
Source: Microbiol Spectr. 2023 Oct 20;11(6):e02426-23. doi: 10.1128/spectrum.02426-23 (PMC10715207; doi:10.1128/spectrum.02426-23)
Supplement: Fig. S1 — Cellulase activity was determined on CMC medium, phosphate solubilization on Pikovskaya's medium, siderophore production on CAS medium, and extracellular protease activity on skim milk medium. [file spectrum.02426-23-s0001.pdf]

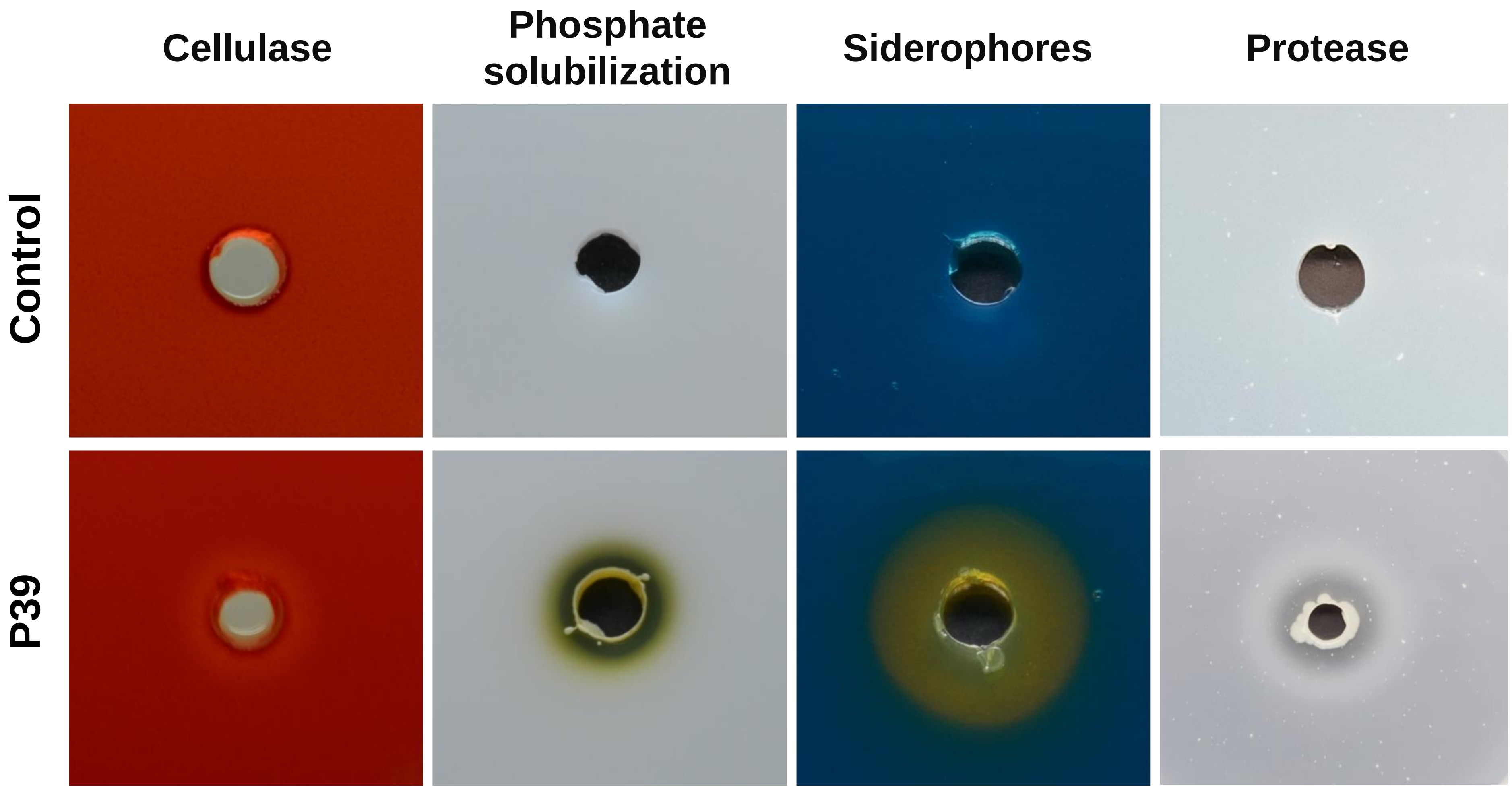

Figure S1. Cellulase activity was determined on CMC medium, phosphate solubilization on Pikovskaya's medium, siderophore production on CAS medium, and extracellular protease activity on skim milk medium. The tested isolate, *Paraburkholderia busanensis* sp. nov. P39, showed weakly positive cellulase activity, positive phosphate solubilization, siderophore production, and protease activity.
