# Supplementary material for: Taxonomy-guided selection of Paraburkholderia busanensis sp. nov.: a versatile biocontrol agent with mycophagy against Colletotrichum scovillei causing pepper anthracnose
Source: Microbiol Spectr. 2023 Oct 20;11(6):e02426-23. doi: 10.1128/spectrum.02426-23 (PMC10715207; doi:10.1128/spectrum.02426-23)
Supplement: Table S1 — List of the isolated strains and the accession numbers of their 16S rRNA partial sequences on NCBI GenBank database. [file spectrum.02426-23-s0002.docx]

**Table S1** List of the isolated strains and the accession numbers of their 16S rRNA partial sequences on NCBI GenBank database

| **No.** | **Isolated strain** | **Acession number** | **No.** | **Isolated strain** | **Acession number** | **No.** | **Isolated strain** | **Acession no.** | **No.** | **Isolated strain** | **Acession no.** | **No.** | **Isolated strain** | **Acession no.** | **No.** | **Isolated strain** | **Acession no.** |
| --- | --- | --- | --- | --- | --- | --- | --- | --- | --- | --- | --- | --- | --- | --- | --- | --- | --- |
| 1 | 16-P1 | OQ771964 | 36 | 16-P57 | OQ771996 | 71 | 16-P129 | OQ772029 | 106 | 16-E86 | OQ772063 | 141 | 16-E202 | OQ772097 | 176 | 17-P48 | OQ772131 |
| 2 | 16-P2 | OQ771965 | 37 | 16-P58 | OQ771997 | 72 | 16-P130 | OQ772030 | 107 | 16-E95 | OQ772064 | 142 | 16-E203 | OQ772098 | 177 | 17-P46 | OQ772132 |
| 3 | 16-P3 | OQ771966 | 38 | 16-P61 | OQ771998 | 73 | 16-P132 | OQ772031 | 108 | 16-E104 | OQ772065 | 143 | 16-E205 | OQ772099 | 178 | 17-P45 | OQ772133 |
| 4 | 16-P5 | OQ771967 | 39 | 16-P63 | OQ771999 | 74 | 16-P133 | OQ772032 | 109 | 16-E105 | OQ772066 | 144 | 17-P84 | OQ772100 | 179 | 17-P100 | OQ772134 |
| 5 | 16-P6 | OQ771968 | 40 | 16-P71 | OQ772000 | 75 | 16-P134 | OQ772033 | 110 | 16-E106 | OQ772067 | 145 | 17-P83 | OQ772101 | 180 | 17-P82 | OQ772135 |
| 6 | 16-P7 | OQ771969 | 41 | 16-P74 | OQ772001 | 76 | 16-P135 | OQ772034 | 111 | 16-E107 | OQ772068 | 146 | 17-P75 | OQ772102 | 181 | 17-P81 | OQ772136 |
| 7 | 16-P8 | OQ771970 | 42 | 16-P77 | OQ772002 | 77 | 16-P138 | OQ772035 | 112 | 16-E113 | OQ772069 | 147 | 17-P74 | OQ772103 | 182 | 17-P80 | OQ772137 |
| 8 | 16-P10 | OQ771971 | 43 | 16-P82 | OQ772003 | 78 | 16-P149 | OQ772036 | 113 | 16-E117 | OQ772070 | 148 | 17-P72 | OQ772104 | 183 | 17-P79 | OQ772138 |
| 9 | 16-P12 | OQ771972 | 44 | 16-P83 | OQ772004 | 79 | 16-P150 | OQ772037 | 114 | 16-E118 | OQ772071 | 149 | 17-P71 | OQ772105 | 184 | 17-P78 | OQ772139 |
| 10 | 16-P13 | OQ771973 | 45 | 16-P84 | OQ772005 | 80 | 16-P151 | OQ772038 | 115 | 16-E122 | OQ772072 | 150 | 17-P70 | OQ772106 | 185 | 17-P77 | OQ772140 |
| 11 | 16-P14 | OQ771974 | 46 | 16-P85 | OQ772006 | 81 | 16-P152 | OQ772039 | 116 | 16-E123 | OQ772073 | 151 | 17-P69 | OQ772107 | 186 | 17-P76 | OQ772141 |
| 12 | 16-P18 | OQ771975 | 47 | 16-P86 | OQ772007 | 82 | 16-P153 | OQ772040 | 117 | 16-E124 | OQ772074 | 152 | 17-P68 | OQ772108 | 187 | 17-P67 | OQ772142 |
| 13 | 16-P20 | OQ781149 | 48 | 16-P87 | OQ772008 | 83 | 16-P154 | OQ772041 | 118 | 16-E129 | OQ772075 | 153 | 17-P64 | OQ772109 | 188 | 17-P66 | OQ772143 |
| 14 | 16-P19 | OQ771976 | 49 | 16-P88 | OQ772009 | 84 | 16-P156 | OQ772042 | 119 | 16-E133 | OQ772076 | 154 | 17-P63 | OQ772110 | 189 | 17-P65 | OQ772144 |
| 15 | 16-P21 | OQ771977 | 50 | 16-P89 | OQ772010 | 85 | 16-P157 | OQ772043 | 120 | 16-E136 | OQ772077 | 155 | 17-P62 | OQ772111 | 190 | 17-P52 | OQ772145 |
| 16 | 16-P23 | OQ771978 | 51 | 16-P101 | OQ772011 | 86 | 16-E20 | OQ772044 | 121 | 16-E140 | OQ772078 | 156 | 17-P61 | OQ772112 | 191 | 17-P50 | OQ772146 |
| 17 | 16-P25 | OQ771979 | 52 | 16-P103 | OQ772012 | 87 | 16-E29 | OQ772045 | 122 | 16-E145 | OQ772079 | 157 | 17-P60 | OQ772113 | 192 | 17-P47 | OQ772147 |
| 18 | 16-P28 | OQ771980 | 53 | 16-P104 | OQ772013 | 88 | 16-E32 | OQ772046 | 123 | 16-E149 | OQ772080 | 158 | 17-P59 | OQ772114 | 193 | 17-P44 | OQ772148 |
| 19 | 16-P29 | OQ771981 | 54 | 16-P106 | OQ772014 | 89 | 16-E34 | OQ772047 | 124 | 16-E151 | OQ772081 | 159 | 17-P58 | OQ772115 | 194 | 17-P38 | OQ772149 |
| 20 | 16-P30 | OQ771982 | 55 | 16-P107 | OQ772015 | 90 | 16-E35 | OQ772048 | 125 | 16-E153 | OQ772082 | 160 | 17-P57 | OQ772116 | 195 | 17-P21 | OQ772150 |
| 21 | 16-P32 | OQ771983 | 56 | 16-P108 | OQ772016 | 91 | 16-E36 | OQ772049 | 126 | 16-E157 | OQ772083 | 161 | 17-P56 | OQ772117 | 196 | 17-P19 | OQ772151 |
| 22 | 16-P33 | OQ771984 | 57 | 16-P109 | OQ772017 | 92 | 16-E40 | OQ772050 | 127 | 16-E160 | OQ772084 | 162 | 17-P55 | OQ772118 | 197 | 17-P18 | OQ772152 |
| 23 | 16-P34 | OQ771985 | 58 | 16-P111 | OQ772018 | 93 | 16-E44 | OQ772051 | 128 | 16-E163 | OQ772085 | 163 | 17-P54 | OQ772119 | 198 | 17-P17 | OQ772153 |
| 24 | 16-P39 | OQ771986 | 59 | 16-P114 | OQ772019 | 94 | 16-E45 | OQ772052 | 129 | 16-E165 | OQ772086 | 164 | 17-P53 | OQ772120 | 199 | 17-P16 | OQ772154 |
| 25 | 16-P41 | OQ771987 | 60 | 16-P115 | OQ772020 | 95 | 16-E46 | OQ772053 | 130 | 16-E170 | OQ772087 | 165 | 17-P43 | OQ772121 | 200 | 17-P15 | OQ772155 |
| 26 | 16-P43 | OQ771988 | 61 | 16-P117 | OQ772021 | 96 | 16-E49 | OQ772054 | 131 | 16-E175 | OQ772088 | 166 | 17-P42 | OQ772122 | 201 | 17-P14 | OQ772156 |
| 27 | 16-P45 | OQ771989 | 62 | 16-P118 | OQ772022 | 97 | 16-E53 | OQ772055 | 132 | 16-E181 | OQ772089 | 167 | 17-P41 | OQ772123 | 202 | 17-P13 | OQ772157 |
| 28 | 16-P46 | OQ771990 | 63 | 16-P120 | OQ772023 | 98 | 16-E57 | OQ772056 | 133 | 16-E183 | OQ772090 | 168 | 17-P40 | OQ772124 | 203 | 17-P12 | OQ772158 |
| 29 | 16-P47 | OQ771991 | 64 | 16-P122 | OQ772024 | 99 | 16-E61 | OQ772057 | 134 | 16-E185 | OQ772091 | 169 | 17-P39 | OQ772125 | 204 | 17-P11 | OQ772159 |
| 30 | 16-P48 | OQ771992 | 65 | 16-P123 | OQ772025 | 100 | 16-E66 | OQ772058 | 135 | 16-E190 | OQ772092 | 170 | 17-P22 | OQ772126 | 205 | 17-P10 | OQ772160 |
| 31 | 16-P52 | OQ771993 | 66 | 16-P124 | OQ772026 | 101 | 16-E69 | OQ772059 | 136 | 16-E191 | OQ772093 | 171 | 17-P20 | OQ772127 | 206 | 17-P39 | OQ772166 |
| 32 | 16-P53 | OQ771994 | 67 | 16-P125 | OQ772027 | 102 | 16-E72 | OQ772060 | 137 | 16-E193 | OQ772094 | 172 | 17-P7 | OQ772162 |  |  |  |
| 33 | 16-P56 | OQ771995 | 68 | 16-P128 | OQ772028 | 103 | 16-E84 | OQ772061 | 138 | 17-P8 | OQ772161 | 173 | 17-P9 | OQ772128 |  |  |  |
| 34 | 17-P4 | OQ772165 | 69 | 17-P5 | OQ772164 | 104 | 17-P6 | OQ772163 | 139 | 16-E198 | OQ772095 | 174 | 17-P51 | OQ772129 |  |  |  |
| 35 | 17-P2 | OQ772167 | 70 | 17-P1 | OQ772168 | 105 | 16-E85 | OQ772062 | 140 | 16-E200 | OQ772096 | 175 | 17-P49 | OQ772130 |  |  |  |
